# A simple method for K-wire capping using nelaton rubber catheter

**DOI:** 10.4103/0970-0358.53027

**Published:** 2009

**Authors:** Jung-Woo Hu, Sung-No Jung

**Affiliations:** Department of Plastic Surgery, Uijongbu St. Mary's Hospital, College of Medicine, Catholic University of Korea, 65-1 Kumoh-Dong, Uijongbu, 480-135, Korea

Sir,

Comminuted or complex intra-articular fractures of the finger are difficult to treat and the optimal treatment has not been established yet. In these cases, we have used two syringe caps of the same size and two K-wires as an external fixator.[1,2]

**Figure 1:**
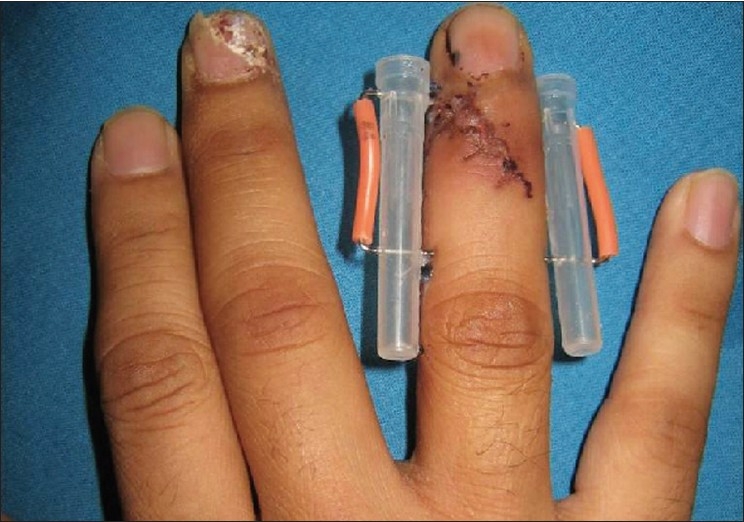
Nelaton rubber catheter as a protection for the external fixator

We noticed that the ends of K-wires which were fixated to the bone could cause injury to the skin of the adjacent finger. Moreover, bare wires could migrate, and cause infection of the pinning site. Therefore, we used the nelaton rubber catheter to reduce pin migration, infection of the pinning site, and trauma due to the sharp tip of the wire in th e situation of applying the external fixator. The external fixator is composed of two syringe caps as the cross bar, and two K-wires which hold the syringe caps. K-wires are inserted, one proximal and one distal to the fracture. First one K-wire is passed through a syringe cap, then through the bone proximal to the fracture site and finally after coming out of the bone and skin on the opposite side, it is passed through the second syringe cap. After reduction, the other K wire is inserted in the same way as above, this time distal to the fracture site. The exposed portion of the wire is bent at an angle of about 90 degrees. At this time we use nelaton rubber catheters (No.4 French) for covering wire tips of the external fixator [Figure 1]. The method, which we have developed and presented herein, is novel and simple. It has also proven its reliability and effectiveness. We have treated 32 patients with comminuted or complex intra-articular fracture, using this technique between March 2004 and December 2006. After the use of nelaton catheter, there were no complications of infection or pin migration, or scratching wound caused by sharp K-wire tip. Care must be taken to remove particles from the syringe cap produced while piercing it. These particles can enter the skin with the next pass of the K-wire tip and result in foreign body reaction.

We recommend the nelaton rubber catheter for protection of the adjacent fingers from the sharp K-wire tips of the external fixator and for prevention of pin migration and infection. To conclude, this is an inexpensive and simple technique where the hardware can be easily procured.
